# Microbiota changes: the unseen players in cervical cancer progression

**DOI:** 10.3389/fmicb.2024.1352778

**Published:** 2024-02-08

**Authors:** W. M. Fong Amaris, Paulo Pimentel de Assumpção, Leonardo Jacomo Valadares, Fabiano Cordeiro Moreira

**Affiliations:** ^1^Instituto de Ciências Biológicas, Universidade Federal do Pará, Belém, Brazil; ^2^Núcleo de Pesquisas em Oncologia, Universidade Federal do Pará, Belém, Brazil; ^3^Hospital Universitário João de Barros Barreto, Universidade Federal do Pará, Belém, Brazil

**Keywords:** microbiota changes, cervical cancer, progression, biomarkers, vaginal microbiota

## Abstract

Cervical cancer ranks among the most prevalent cancers globally with high-risk human papillomaviruses implicated in nearly 99% of cases. However, hidden players such as changes in the microbiota are now being examined as potential markers in the progression of this disease. Researchers suggest that changes in the vaginal microbiota might correlate with cervical cancer. This review provides a comprehensive look at the microbiota changes linked with the advancement of cervical cancer. It also scrutinizes the databases from past studies on the microbiota during healthy and cancerous stages, drawing connections between prior findings concerning the role of the microbiota in the progression of cervical cancer. Preliminary findings identify *Fusobacterium* spp., *Peptostreptococcus* spp., *Campylobacter* spp., and *Haemophilus* spp., as potential biomarkers for cervical cancer progression. *Alloscardovia* spp., *Eubacterium* spp., and *Mycoplasma* spp. were identified as potential biomarkers for HPVs (+), while *Methylobacterium* spp. may be indicative of HPV (−). However, the study’s limitations, including potential biases and methodological constraints, underscore the need for further research to validate these findings and delve deeper into the microbiota’s role in HPV development. Despite these limitations, the review provides valuable insights into microbiota trends during cervical cancer progression, offering direction for future research. The review summarizes key findings from previous studies on microbiota during healthy and cancerous stages, as well as other conditions such as CIN, SIL, HPV (+), and HPV (−), indicating a promising area for further investigation. The consistent presence of HPV across all reported cervical abnormalities, along with the identification of distinct bacterial genera between cancerous and control samples, suggests a potential link that merits further exploration. In conclusion, a more profound understanding of the microbial landscape could elucidate the pathogenesis of cervical diseases and inform future strategies for diagnosis, prevention, and treatment.

## 1 Introduction

Cervical cancer (CAN) is a significant health concern for women worldwide, ranking as one of the most common cancers (Arbyn et al., 2020; WHO, 2020; Aobchey et al., 2022; Wang et al., 2022). As per the World Health Organization (WHO), in 2018, approximately 570,000 women were diagnosed with cervical cancer globally, resulting in roughly 311,000 fatalities (WHO, 2020; Wickramasinghe et al., 2021; Zhang et al., 2022). Furthermore, the WHO projected in 2020 that the annual incidence of new cases of this disease could rise from 570,000 to 700,000 between 2018 and 2030 (WHO, 2020).

The prevalence of this disease is notably higher in low-to middle-income countries, nonetheless, it affects women globally. The number of deaths in low and middle-income countries accounted for an estimated 90% of the 311,000 global fatalities. Consequently, age-standardized incidence rates fluctuate from 75 per 100,000 women in the highest-risk countries to fewer than 10 per 100,000 women in the lowest-risk countries (Bray et al., 2018; WHO, 2020). This disparity underscores the urgency to address cervical cancer, particularly in regions with higher risk factors.

Human papillomavirus (HPV) infection is pivotal in the development of cervical cancer, with nearly 99% of cases associated with high-risk HPV strains (WHO, 2020; Jiang and Wang, 2022). However, other contributing factors such as tobacco use, immunosuppression, malnutrition, and low socioeconomic status are also implicated (ACCP, 2004; Ghebre et al., 2017; Zhang et al., 2020). Persistent infection with various types of HPV is acknowledged as a contributing factor in the progression of cervical intraepithelial neoplasia (CIN) and invasive cervical cancer (ICC). Nevertheless, the complete involvement of HPV in the entire tumorigenic process remains a topic of ongoing debate due to insufficient data (Muñoz, 2000; Castellsagué, 2008; Wheeler, 2013; Kori and Arga, 2018; So et al., 2020; Kang et al., 2021).

Recent literature posits an intriguing hypothesis: microorganisms may play a significant role in malignancies. This theory suggests that there could be unexplored mechanisms during infections where these microscopic entities take a leading role (Parkin, 2006; Godoy-Vitorino et al., 2018).

This perspective necessitates a broader understanding of the microbial world and its potential influence on disease processes. The interplay between HPV and other microorganisms could add another layer of complexity to the etiology of cervical cancer, warranting further investigation. This new viewpoint not only challenges our current knowledge but also paves the way for innovative research directions in cervical cancer pathogenesis.

The detection of microbial diversity, first accomplished in 1677 by Van Leeuwenhoek through microscopic observation, has evolved significantly over time (Wei et al., 2021). In cervical cancer diagnostics, the Papanicolaou smear, a microscopic biopsy image analysis, has traditionally been the primary modality (Long et al., 2017; Kori and Arga, 2018). However, its reliability is debatable due to its dependence on human interpretation (Long et al., 2017). Despite several alternate cervical cancer screening methods proposed over the years such as cytological testing alone, standalone hrHPV testing, and cytological + hrHPV combination testing (co-testing) (Curry et al., 2018; Kim et al., 2018; Terasawa et al., 2022), the 5 years survival rate remains a dismal 66% (Long et al., 2017; Basic et al., 2021; Qu et al., 2021; Hou et al., 2022).

Treatment strategies for cervical cancer, such as surgical resection, radiotherapy, and chemotherapy, are frequently challenged by tumor metastasis and recurrence, complicating disease management (Mallmann and Mallmann, 2016; Vordermark, 2016; Koh et al., 2019; Li et al., 2021). Further, patients often suffer from side effects related to these treatments. This highlights a significant problem: the urgent need for novel, reliable diagnostic methods for cervical cancer that can improve early detection and thereby enhance survival rates (Zhu et al., 2016; Long et al., 2017; Liu et al., 2018; Koh et al., 2019; Han et al., 2021).

The exploration of microbial diversity has been significantly enhanced by advancements in culture technologies. However, due to inherent challenges associated with laboratory culturing procedures, our understanding is not yet exhaustive (Wei et al., 2021). In response to this, techniques centered around molecular sample analysis have emerged within the field of omics, paving the way for a more detailed investigation of microbial diversity (Wei et al., 2021).

The advent of laboratory automation has facilitated the deployment of high-throughput-omics technologies. These sophisticated methodologies enable an in-depth characterization of samples collected from both patients and healthy individuals, thereby expanding our knowledge of microbial ecosystems. One such transformative innovation is next-generation sequencing (NGS). This technique has unlocked the potential to delineate the intricate complexity of microbial communities and human microbiota, providing valuable insights into the influence of the microbiome on human health and disease pathologies (Peterson et al., 2009).

Among the various omic approaches, metagenomics stands out for its ability to divulge specific information about the genomes and genes within a microbial community. It serves as an essential first step in microbiome studies (Marchesi and Ravel, 2015; Aguiar-Pulido et al., 2016). The primary goal of metagenomics is to determine the taxonomic profile of a microbial community, typically involving NGS post-DNA extraction from samples, followed by assembly or mapping to a reference database, and subsequent annotation (Marchesi and Ravel, 2015; Aguiar-Pulido et al., 2016). This method has become particularly prevalent in investigating the microbial composition within the vaginal environment.

The female genital tract serves as a critical ecological niche for human microbiota (Gao et al., 2013), housing *Lactobacillus* species that contribute to metabolic processes, immunological responses, and overall gynecological health (Kang et al., 2021). Known for probiotic benefits, *Lactobacillus* species help combat vaginal dysbiosis (Machado et al., 2022; Pacha-Herrera et al., 2022; Rodríguez-Arias et al., 2022). Detailed insights follow in this review’s upcoming sections. There is an emerging body of literature suggesting that alterations in the vaginal microbiota may be linked to cervical cancer (Klein et al., 2020b; Norenhag et al., 2020; So et al., 2020; Tango et al., 2020; Kang et al., 2021; Sims et al., 2021; Wu et al., 2021; Zhou et al., 2021). Furthermore, numerous studies propose that the vaginal microbiota could play a crucial role in defending women against infections such as HPV, vulvovaginal candidiasis, and other sexually transmitted diseases (Liu et al., 2013; Lewis et al., 2017; Arroyo Mühr et al., 2021; Kang et al., 2021). As such, the cervical microbiota could potentially serve as a biomarker for assessing the risk of cancer progression (Mitra et al., 2016a,b; Curty et al., 2019; Arroyo Mühr et al., 2021).

The exploration of the human microbiome has been an exciting journey, with techniques evolving from 16S sequencing (Audirac-Chalifour et al., 2016; Dareng et al., 2016; di Paola et al., 2017; Klein et al., 2020b; Norenhag et al., 2020; So et al., 2020; Tango et al., 2020; Sims et al., 2021; Wu et al., 2021; Zhou et al., 2021), PCR (Norenhag et al., 2020), and microarray (Borgdorff et al., 2014; Norenhag et al., 2020) to cutting-edge methods like RNA-seq (Kori and Arga, 2018; Klein et al., 2020a; Chang et al., 2021) and Whole Genome Shotgun (WGS) (Klein et al., 2020a; Wei et al., 2021). A significant milestone in this journey was the commencement of the Human Microbiome Project (HMP) in 2008, which aimed to map the microbial landscape across various body parts, including the lower genital tract of healthy individuals (Castanheira et al., 2021). From this wealth of research, a startling revelation has emerged: approximately 20% of all fatal cancers are microbially induced (Godoy-Vitorino et al., 2018). Moreover, numerous studies have drawn significant correlations between alterations in the microbiome and cancer phenotypes (Elinav et al., 2019; Poore et al., 2020; Banavar et al., 2021). This underlines the potential of the microbiota as a treasure trove of biomarkers that could revolutionize clinical diagnostics and disease management.

This review is an ambitious endeavor to chart the intricate relationship between the microbiota and cervical cancer progression. We delve into the diverse universe of microorganisms implicated in cervicovaginal dysbiosis, providing an authoritative synthesis of prior research on both CONTROL (healthy) samples and CAN stage. Our goal is to offer an updated perspective on the role of microbiota in cervical cancer progression, thereby filling a crucial gap in the existing literature.

While our analysis provides a comprehensive overview based on the data available at the time of our research, it’s crucial to recognize the fluidity and rapid evolution of scientific knowledge. As such, newer developments may not have been captured. This underscores the need for ongoing research in this field. Therefore, we strongly advocate for broader studies using metagenomics and metatranscriptomics, as these techniques hold immense promise in untangling the intricate role of microbiota in cervical cancer progression. By deepening our understanding of this critical issue, we can pave the way for innovative therapeutic interventions, heralding a new era in women’s health management.

## 2 Cervical cancer and HPV

According to the World Health Organization (WHO, 2020), the principal instigator of cervical pre-cancer and squamous cervical cancer is the asymptomatic, persistent or chronic infection with one or more high-risk HPV types. While over 100 HPV types have been identified, only a fraction are associated with cervical cancer. Indeed, two specific types, HPV 16 and 18, are implicated in approximately 70% of all reported cervical cancer cases (Pappa et al., 2018; Cohen et al., 2019; Lin et al., 2019; WHO, 2020). Other high-risk HPV types, such as 31, 33, 45, and 58, are less frequently linked to cervical cancer, with prevalence varying by geographic location. Additionally, low-risk HPV types 6 and 11, although not contributing to cervical cancer, are responsible for most genital warts or condylomas (WHO, 2014).

The role of genetic variation in cervical cancer has been underscored by genome-wide association studies (GWAS). Lin et al. (2019), reported that cervical cancer harbors genetic variations across multiple susceptibility loci (Bahrami et al., 2018; Lin et al., 2019). The viral oncoproteins E6 and E7 appear to play a pivotal role in HPV-infected cervical cancers. Integration of the viral genome into the host DNA results in the upregulation of E6 and E7, leading to the deregulation of key proteins within cellular signaling pathways, including the inhibition of two vital tumor suppressor proteins, p53 and pRb (Oyervides-Muñoz et al., 2018; Lin et al., 2019). The combined effect of E6 and E7 viral proteins triggers the process of immortalization in HPV-infected cells. This precedes the malignant metamorphosis of these cells (Da Silva et al., 2021).

Furthermore, Lau et al. (2015) revealed that DNA tumor virus oncogenes, including E7, can bind to and suppress the cGAS-STING DNA-sensing pathway (Lau et al., 2015; Lin et al., 2019). However, it’s worth noting that not all integrations necessarily rely on the expression of the E6 and E7 oncogenes (Groves and Coleman, 2015; Lin et al., 2019). In addition to these findings, several reports have identified driver mutations in cervical cancer, such as PIK3CA (phosphatidylinositide 3-kinases catalytic subunit α), a central protein in the PI3K pathway, KRAS (Kirsten rat sarcoma viral oncogene homolog), and EGFR (epidermal growth factor receptor) (Lin et al., 2019).

## 3 Vaginal microbiota

The vaginal microbiota is a critical component of women’s health (Wu et al., 2021). This complex ecosystem, which operates in harmony with the host, provides protective mechanisms against dysbiosis and infection (Klein et al., 2020a). The function of the vaginal mucosa as a barrier against pathogens is facilitated by the interaction of epithelial cells, the immune system, and various microorganisms (Borgdorff et al., 2016; Taddei et al., 2018; Castanheira et al., 2021).

Dominating this ecosystem are *Lactobacillus* species, which play a significant role in maintaining vaginal health. By producing lactic acid, these bacteria sustain a low pH environment in the cervicovaginal setting, thereby preventing the colonization of harmful opportunistic pathogens, preserving the cervical epithelial barrier, and impeding mucin degradation (Amabebe and Anumba, 2018; Klein et al., 2020a; Norenhag et al., 2020; Salinas et al., 2020; So et al., 2020; Kang et al., 2021; Wu et al., 2021).

However, the composition of the vaginal microbiota is not static. It can be influenced by numerous factors such as genetics, diet, lifestyle, hygiene practices, ethnicity, reproductive age, infections, male factor, usage of antibiotics and contraceptives, sexual activity, physiological status, pregnancy and estrogen levels (Mitra et al., 2016b; Kwasniewski et al., 2018; Wu et al., 2021; Zhou et al., 2021; Baud et al., 2023).

Thanks to new molecular techniques, over 50 microbial species have been identified within the vaginal microbiota, with *Lactobacillus* spp. being the most prevalent (Norenhag et al., 2020; Wu et al., 2021). Among them, *L. crispatus*, *L. gasseri*, *L. inners*, and *L. jensenii* are the most commonly found (Wu et al., 2021).

Further research about the vaginal microbiota in healthy women from different ethnic groups (White, Black, Hispanic and Asian) by Ravel et al. (2011) led to the classification of vaginal bacterial communities into five distinct “community state types” (CST). In this classification, *Lactobacillus* species dominated groups I, II, III, and V. Group IV, on the other hand, was characterized by a diverse set of anaerobic bacteria, including bacteria like *Prevotella* spp., *Streptococcus* spp., *Dialister* spp., *Fannyhessea* spp. (previously known as *Atopobium*), *Gardnerella* spp., *Megasphaera* spp., *Peptoniphilus* spp., *Sneathia* spp., *Eggerthella* spp., *Aerococcus* spp., *Finegoldia* spp., and *Mobiluncus* spp. These findings were consistent with previous research employing 16S rRNA genes (Srinivasan and Fredricks, 2008; Zhou et al., 2010).

Despite the diversity in bacterial species, a commonality across all CST groups was the presence of lactic acid-producing bacteria, suggesting a conserved function throughout these communities.

## 4 Vaginal microbiota and cervical cancer

The human body is a dynamic ecosystem for a myriad of microbes, collectively known as the microbiome. This microbiome plays a pivotal role in maintaining normal bodily functions, including immune modulation and overall protection (Wei et al., 2021; Zhou et al., 2021). Over time, evidence has emerged highlighting the connection between the microbiome, inflammation, and the development and progression of cancer. According to Zhou et al. (2021) and Wei et al. (2021), disturbances in microbial homeostasis can trigger a cascade of immune responses. Chronic inflammation, a byproduct of such disruptions, is a known carcinogenic factor, heightening the host’s susceptibility to cancer (Zhou et al., 2021).

Given the profound implications of the microbiome on health and disease, specifically cervical cancer, advanced research is warranted. The advent of laboratory automation and high-throughput technologies has revolutionized our understanding of microbiome diversity and its potential impacts (Wei et al., 2021). There is mounting scientific evidence pointing towards a correlation between microbiota and cervical cancer (Castanheira et al., 2021; Kang et al., 2021; Wei et al., 2021; Wu et al., 2021; Zhou et al., 2021).

Cervicitis, or inflammation of the cervix, can stem from various conditions, including microbial infections. Chronic cervicitis has been linked to the development of cervical cancer. Pelvic inflammatory disease (PID) in women is typically triggered by ascending bacterial infections from the cervix to the uterus and fallopian tubes. Bacterial vaginosis (BV), a condition characterized by dysbiosis of cervicovaginal bacteria, is also associated with cervicitis. Notably, the microenvironment fostered by BV is reported to facilitate persistent HPV infection, a known precursor to cervical cancer (Castanheira et al., 2021; Zhou et al., 2021).

Various microorganisms, including *Fusobacterium* spp., *Mycoplasma genitalium*, *Chlamydia trachomatis*, *Sneathia* spp., *Anaerococcus* spp., *Peptostreptococcus* spp., *Gardnerella* spp., *Prevotella* spp., *Fannyhessea* spp., *Streptococcus* spp., *Dialister* spp., *Megasphaera* spp., *Peptoniphilus* spp., *Finegoldia* spp., *Mobiluncus* spp. and *Lactobacillus iners* have been implicated in the onset of cervical cancer. Interestingly, *L. iners* is found more frequently in infected women compared to their healthy counterparts. Table 1 provides a detailed overview of these microorganisms and their association with cervical cancer.

**Table 1:** Association between microorganisms and cervical cancer.

Microorganisms	Relationship with cervical cancer	References
*Fusobacterium* spp.	*Fusobacterium* spp., inclusive of *Sneathia* spp., is implicated in creating an immunosuppressive microenvironment characterized by anti-inflammatory cytokines. It plays a significant role in the development of cervical cancer.	Audirac-Chalifour et al. (2016) and Zhou et al. (2021)
	Identified as a microbial biomarker for HPV infection.	Audirac-Chalifour et al. (2016) and Zhou et al. (2021)
	*Sneathia* spp., from the *Fusobacterium* genus, has associations with HPV, cervical intraepithelial neoplasia (CIN), and cervical cancer (CAN). Produces FadA, a virulence factor disrupting the cervical cancer signaling pathway. Overexpression of FadA gene is observed in CAN patients.	Audirac-Chalifour et al. (2016), Mitra et al. (2016b) and Wu et al. (2021)
	Increased presence of *Fusobacterium* spp., may lead to local immunosuppression, promoting HPV immune evasion and disease progression.	Mitra et al. (2016b)
	Distinctly higher levels of *Fusobacterium* spp. in the CAN group. Identified as a marker for both CAN and high-grade squamous intraepithelial lesions (HSIL) groups. May contribute to CAN pathogenesis through chronic inflammation, antiapoptotic activity, or production of carcinogenic substances.	Norenhag et al. (2020), Sims et al. (2021) and Wu et al. (2021)
	The presence of *F. necrophorum* is specifically reported in CAN cases	Audirac-Chalifour et al. (2016), So et al. (2020) and Castanheira et al. (2021)
*Mycoplasma genitalium*	*M. genitalium* is an independent pathogenic microorganism causing a series of intracellular infections. It disrupts tight junctions from epithelial cells, which can lead to bacterial vaginosis (BV) and cervicitis. It increases the incidence of cervical lesions. Some studies suggest that *M. genitalium* can induce chromosomal damage in cells, potentially leading to the formation of cancerous cells. Both *M. genitalium* and *M. hominis* are common mycoplasmas found in female tract infections. These mycoplasmas have been identified in patients with cervicitis and BV. There is a documented association between *M. genitalium* and BV.	Klein et al. (2020a) and Zhou et al. (2021)
*Chlamydia trachomatis*	*C. trachomatis* has been identified as a co-factor for cervical cancer (CAN) development in epidemiologic studies.	Audirac-Chalifour et al. (2016)
	This microorganism can damage the cervical mucosal barrier, facilitating high-risk HPV (hr-HPV) infection of the cervical epithelium. It may induce chronic inflammation and influence local and cellular immunity of the cervix, inhibiting HPV clearance. The persistence of HPV infection, facilitated by *C. trachomatis*, could lead to cervical cancer. Some research suggests that non-bacterial components might affect CAN progression, but there is a lack of extensive studies on this topic.	Zhou et al. (2021)
	It has been associated with cervicitis, the persistence of HPV infection, and BV. Meta-analyses have reported a positive correlation between cervical HPV infection and BV. HPV is considered a primary factor responsible for CAN development.	Kwasniewski et al. (2018) and Klein et al. (2020a)
*Sneathia* spp.	*Sneathia* spp. is a potential microbiological marker of HPV infection.	Mitra et al. (2016b) and Zhou et al. (2021)
	It has a significant association with Bacterial Vaginosis (BV) infection.	Mitra et al. (2016b)
	Reports indicate its presence in cervical intraepithelial neoplasia (CIN) stages 1, 2, and 3.	So et al. (2020)
	Identified as a marker genus of high-grade squamous intraepithelial lesions (HSIL) group.	Wu et al. (2021)
	*S. sanguinegens* is associated with high-grade CIN, whereas *S. amnii* (previously named *Leptotrichia amnionii*) has been linked with cervical cancer, but not HPV infection or CIN.	Mitra et al. (2016b)
	A documented correlation exists between colonization with *S. amnii* and cervical cancer in HPV-positive subjects. *S. amnii* has also been reported as a reliable predictor of BV.	Audirac-Chalifour et al. (2016)
*Gardnerella* spp.	*Gardnerella* spp., particularly *G. vaginalis*, is proposed as a molecular marker due to its role in biofilm formation, which may contribute to the persistence of HPV infection. *Gardnerella* spp. and *Streptococcus* spp. may serve as biomarkers to potentially distinguish invasive cervical cancer (ICC) from cervical intraepithelial neoplasia (CIN), indicating possible disease progression. *Gardnerella* spp. has been associated with bacterial vaginosis (BV) and HPV infection. It is a representative genus in the CIN group and is reported as a biomarker to differentiate patients with CIN from healthy individuals. The presence of *Gardnerella* spp. has been reported in patients from high-grade squamous intraepithelial lesions (HSIL + HPV +) and Low-grade squamous intraepithelial lesions (LSIL) groups. An enrichment of anaerobic bacteria like *G. vaginalis* has been described in women with CIN and cervical cancer (CAN). This bacterium, a gram-variable facultative anaerobe, becomes more abundant during BV. *G. vaginalis* has been significantly associated with the risk for HSIL, high-grade CIN, and CAN. It was identified as a high risk for developing CIN2, CIN3, and CAN. High levels of *Gardnerella* spp. are common among women persistently infected with hr-HPV for 1 year. Specifically, *G. vaginalis* has been associated with CIN. Another study reported a higher prevalence of *G. vaginalis* in HPV-negative women with non-cervical lesions (NCL), but its presence decreased across the HPV-positive, SIL, and CAN groups.	Audirac-Chalifour et al. (2016), Mitra et al. (2016b), Kwasniewski et al. (2018), Klein et al. (2020a), Norenhag et al. (2020), Castanheira et al. (2021), Kang et al. (2021) and Zhou et al. (2021)
*Dialister* spp.	*Dialister* spp., along with *Prevotella* spp., has been reported as marker genera of the cervical cancer (CAN) group. These are opportunistic pathogens whose activities are influenced by or influence *Lactobacillus* spp. *D. invisus*, a Gram-negative coccobacillus, has been linked to new HPV-type infections within a year in women with typical cytological results. Notably, *D. invisus* has been significantly associated with high-grade squamous intraepithelial neoplasia and an increased risk of CAN	So et al. (2020) and Wu et al. (2021)
*Eggerthella* spp.	*Eggerthella* spp. has been mentioned only once in relation to cervical cancer. There is no available information that directly associates or disassociates it with cervical cancer. It is included in the IV CST (community state type), a classification system for vaginal microbial communities.	Ravel et al. (2011)
*Prevotella* spp.	The abundance of *Prevotella* spp. is associated with HPV persistence and is inversely related to the quantity of *Lactobacillus*. This bacterium may cause infections like bacterial vaginosis (BV) and has been linked with HPV persistence. Notably, *P. bivia*, *P. amnii*, and *P. timonensis* have been reported in HPV positive samples. *Prevotella* spp. and *Lactobacillus* spp. reportedly play an antagonistic role in the progression of squamous intraepithelial lesions (SIL) and cervical cancer (CAN) through NLRs signaling and other pathways. They are reported as marker genera of the CAN group. It’s speculated that these bacteria might drive chronic inflammation and antiapoptotic activity. *Prevotella* spp. is abundant in the cervical intraepithelial neoplasia (CIN) group. Furthermore, three potential biomarkers have been identified: *Lactobacillus* spp., *Gardnerella* spp., and *Prevotella* spp., which can robustly predict and distinguish patients with CIN from healthy individuals. Specifically, *P. buccalis* and *P. timonensis* have been significantly associated with the risk for high-grade squamous intraepithelial lesions (HSIL) and CAN. These species have been reported in the CIN1 group, while *P. disiens* has been reported in the CIN2 or CIN3 groups.	Amabebe and Anumba (2018), So et al. (2020) and Wu et al. (2021)
*Fannyhessea* spp.	A high abundance of *Fannyhessea* spp. in the cervix vaginal microflora may serve as a critical marker for cervical lesions. The dominance of *A. vaginae*, similar to *G. vaginalis*, is particularly noted in cases of bacterial vaginosis and significantly contributes to the risk of developing cervical neoplasia. *F. vaginae* has been reported in cases of cervical intraepithelial neoplasia 2 or 3 (CIN2 or CIN3). Additionally, infection with this bacterium is significantly associated with the risk of developing CIN2, CIN3, and cervical cancer (CAN). Both *G. vaginalis* and *F. vaginae* have been proposed as molecular markers due to their ability to form a biofilm that may contribute to viral persistence.	So et al. (2020) and Kang et al. (2021)
*Streptococcus* spp.	*Streptococcus* spp. species have been related to bacterial vaginosis (BV). They have also been identified in cases of high-grade squamous intraepithelial lesion (HSIL), low-grade squamous intraepithelial lesion (LSIL), and normal controls. Related to aerobic vaginitis (AV), another factor possibly related to CIN. -*Streptococcus* spp. has been reported as a representative genus in the cervical cancer (CAN) group. A study suggested that it could serve as a potential biomarker for distinguishing CAN, possibly through the activation of multiple inflammatory cytokines, and may affect human vaginal and cervical epithelial cells. *Gardnerella* spp., *Streptococcus* spp., *Finegoldia* spp., *Anaerococcus* spp., and *Lactobacillus* spp. are considered the most impactful factors to differentiate CAN from cervical intraepithelial neoplasia (CIN). However, when it comes to distinguishing invasive cervical carcinoma (ICC) from CIN, *Gardnerella* spp. or *Streptococcus* spp. have been reported as potential biomarkers. Specifically, *S. dysgalactiae* has been reported in cases of CAN.	Kwasniewski et al. (2018), Amabebe and Anumba (2020), So et al. (2020), Plisko et al. (2021) and Kang et al. (2021)
*Mobiluncus* spp.	*Mobiluncus* spp. has been reported as one of the organisms causing bacterial vaginosis (BV). This condition occurs when there’s an imbalance in the natural bacteria levels in the vagina, leading to discomfort and pain. There are mainly two species of *Mobiluncus* spp. that have been identified: *M. mulieris* and *M. curtisii*.	Mitra et al. (2016a), Amabebe and Anumba (2018), Kwasniewski et al. (2018) and Klein et al. (2020a)
*Megasphaera* spp.	Marker genera of the CAN (community state type anaerobe non-dominated) group. Mentioned in relation with the SIL (squamous intraepithelial lesion) group, particularly noted for its relative abundance of *M. elsdenii* and presence in the CAN group. *Sneathia* spp., *M. elsdenii*, and *Shuttleworthia satelles* are most representative according to the SIL group. *M. elsdenii* was reported for the first time in women with SIL. *Megasphaera* spp. and *Sneathia amnionii* are considered predictors of bacterial vaginosis (BV).	Audirac-Chalifour et al. (2016) and Wu et al. (2021)
*Peptoniphilus* spp.	Reported as a marker genera of the CAN (community state type anaerobe non-dominated) group. Part of the IV CST (community state type), a classification system for vaginal microbial communities.	Ravel et al. (2011), So et al. (2020) and Wu et al. (2021)
*Aerococcus* spp.	*Aerococcus* spp. has been reported in relation to HPV clearance in CONTROL samples. It is included in the IV CST (community state type), a classification system for vaginal microbial communities.	Ravel et al. (2011)
*Finegoldia* spp.	Reported as a significant factor in distinguishing CAN (community state type anaerobe non-dominated) from CIN (cervical intraepithelial neoplasia). *F. magna* is associated with high-grade squamous intraepithelial neoplasia and CAN risk. It has a significant relationship with the risk of developing CIN2 or CIN3, and CAN. *F. magna* typically appears on the skin and mucous membranes. It is associated with vaginosis.	So et al. (2020) and Kang et al. (2021)
*Lactobacillus jensenii*	Depletion of specific *Lactobacilli* species—*L. crispatus*, *L. gasseri* or *L. jensenii*-is associated with a predisposition towards bacterial vaginosis and other proinflammatory states. This depletion can lead to DNA cell damage and potentially carcinogenic changes. In 20% of CAN (community state type anaerobe non-dominated) cases, there were low levels of *L. jensenii*, which were related to severe lesions. Women with high-grade CIN (cervical intraepithelial neoplasia) had lower levels of *L. jensenii* than those with low-grade CIN. *L. jensenii* and *L. vaginalis* were found only in samples from women with NCL (no cervical lesion).	Audirac-Chalifour et al. (2016), Mitra et al. (2016a), Castanheira et al. (2021) and Sims et al. (2021)
*Lactobacillus gasseri*	*L. gasseri* is reported to potentially be associated with the most rapid clearance of acute HPV infection. It has been proposed as a potential therapeutic species for maintaining cervical health. PCR-based techniques have shown that *L. gasseri* is negatively associated with *L. iners* and *F. vaginae* species, which often co-associate and are suggested to pose an intermediate and high risk for the development of CIN (cervical intraepithelial neoplasia). Depletion of specific *Lactobacilli* species—*L. crispatus*, *L. gasseri* or *L. jensenii*-is associated with a predisposition towards bacterial vaginosis and other proinflammatory states. This depletion can lead to DNA cell damage and potentially carcinogenic changes.	Mitra et al. (2016a), Castanheira et al. (2021) and Sims et al. (2021)
*Lactobacillus crispatus*	The vaginal epithelial mucus layer’s protective function is enhanced, and autophagy activity is observed when *L. crispatus* dominates the vaginal microbiota. Vaginal microbiota dominated by *L. crispatus* is associated with a lower risk of HPV, CIN (cervical intraepithelial neoplasia), and CAN (community state type anaerobe non-dominated) infection. *L. crispatus* is related to maintaining the integrity of the protective mucosal surface layer and poses a lower risk of opportunistic bacterial and viral urogenital infections. The presence of *L. crispatus* has been negatively correlated with CIN. A marked decrease of *L. crispatus* was found in the CIN1, CIN2, CIN3, and CAN groups. *L. crispatus* has been reported as the most protective microorganism against HPV and HIV due to its antimicrobial compound production. Depletion of *L. crispatus* and increased abundance of anaerobic bacteria such as *Gardnerella vaginalis*, *Peptostreptococcus anaerobius*, and *Porphyromonas venonis* is significantly more common in women with CIN and CAN. *L. crispatus* has been reported as the most effective microorganism in preventing bacterial dysbiosis compared to *L. iners*. Evidence suggests that *L. iners* is associated with disease. H_2_O_2_ is thought to be produced by *L. crispatus* rather than *L. iners.* *L. crispatus* produces both D-lactic acid and L-lactic acid.	Audirac-Chalifour et al. (2016), Amabebe and Anumba (2018), Norenhag et al. (2020), So et al. (2020), Castanheira et al. (2021), Sims et al. (2021) and Zhou et al. (2021)
*Lactobacillus iners*	*L. iners* is the most commonly reported *Lactobacillus*-dominated CST (community state type) detected in women diagnosed with CIN (cervical intraepithelial neoplasia). It has a small genome, indicative of a symbiotic or parasitic lifestyle. Some researchers suggest that *L. iners* may have clonal variants that promote health in some cases and are associated with dysbiosis and disease predisposition in others. Microbiomes dominated by *L. iners* are less protective against cervicovaginal infections and exhibit higher rates of HPV infection and cervical dysplasia. *L. iners* does not appear to share many protective mechanisms with other *Lactobacillus* species and is considered intermediate in its ability to prevent cervical disease. Compared to other *Lactobacillus* species, *L. iners* may be less capable of inhibiting the colonization of strict anaerobes and pathobionts. *L. iners* appears more capable of surviving and adapting to a wide range of pH and other metabolic stress-related conditions due to the constitutive and inducible expression of genes not seen in other Lactobacilli. *L. iners*-dominated microbiota is usually associated with dysbiosis and appears less stable and more prone to transition. *L. iners* was more frequently detected in co-infected women than healthy ones. It has been reported in high proportion in women with HSIL (high-grade squamous intraepithelial lesions) and LSIL (low-grade squamous intraepithelial lesions) along with *L. acidophilus* and *L. crispatus*. *L. iners* has been found in women with HIV, HPV, HSV-2 (herpes simplex virus, type 2), CIN, and CAN (community state type anaerobe non-dominated). The presence of *L. iners* has been proposed as a higher risk of HPV, SIL (squamous intraepithelial lesions), and CAN. There’s an association between *L. iners* and CIN or even CAN.	Audirac-Chalifour et al. (2016), Mitra et al. (2016a), Amabebe and Anumba (2018), Kwasniewski et al. (2018), Norenhag et al. (2020), So et al. (2020), Castanheira et al. (2021), Sims et al. (2021) and Zhou et al. (2021)

HC, healthy controls; SIL, squamous intraepithelial lesions; LSIL, low-grade squamous intraepithelial lesions; HSIL, high-grade squamous intraepithelial lesions; CIN, cervical intraepithelial neoplasia; ICC, invasive cervical cancer; CAN, cervical cancer; HPV, human papillomavirus; BV, bacterial vaginitis; PID, pelvic inflammatory disease.

**Figure 1:**
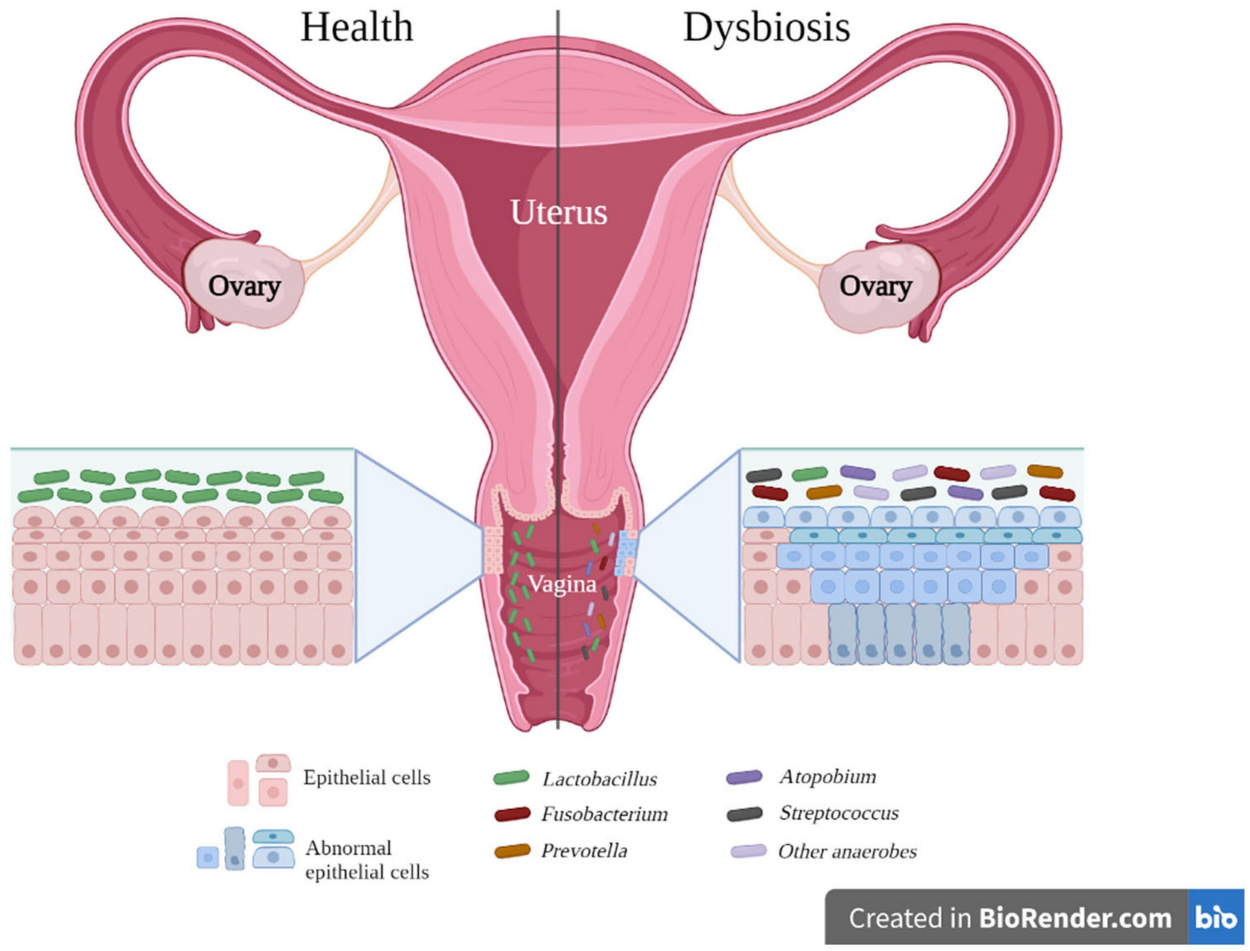
Vaginal microorganisms in a healthy vagina versus a dysbiosis stage in the vagina. Source of image: Authors and adapted from Zhou et al. (2021). The figure reveals the transformation that the vaginal microbiota undergoes between healthy and dysbiotic conditions. In a healthy state, the environment is primarily characterized by the presence of *Lactobacillus* species. On the other hand, in a state of dysbiosis, the environment is largely dominated by a variety of other bacteria, including *Prevotella* spp., the bacterium now known as *Fannyhessea* spp. (formerly *Atopobium* spp.), *Streptococcus* spp., and other anaerobic bacteria.

As illustrated in Table 1, microorganisms such as *Fusobacterium* spp., *Sneathia* spp., *Anaerococcus* spp., *Peptostreptococcus* spp., *Gardnerella* spp., *Prevotella* spp., *Dialister* spp., and *Megasphaera* spp. have been identified as biological markers for cervical cancer (CAN), high-grade squamous intraepithelial lesions (HSIL), and cervical intraepithelial neoplasia (CIN). Conversely, *Lactobacillus crispatus*, *Lactobacillus gasseri*, and *Lactobacillus jensenii* are associated with a decreased risk of infections, including HPV, CIN, and CAN. For an organized overview of this information, please refer to Table 2. This table presents a clear view of the microorganisms found at different disease stages in the vagina. These stages include squamous intraepithelial lesions (SIL)—further divided into low-grade (LSIL) and high-grade (HSIL), cervical intraepithelial neoplasia (CIN), invasive cervical cancer (ICC) or cervical cancer (CAN), and other infections such as pelvic inflammatory disease (PID), HPV, and bacterial vaginitis (BV). Additionally, a column has been included to indicate the microorganisms found in healthy controls (HC). Figure 1 provides a visual representation of the microorganisms present during vaginal inflammation, offering insight into the microbial landscape under these conditions.

**Table 2:** Microorganisms linked to various conditions in the vaginal environment.

HC	SIL		CIN	ICC/CAN	Other conditions (PID, HPV, BV)
	LSIL	HSIL			
*L. crispatus* (Ravel et al., 2011; Mitra et al., 2016b; Amabebe and Anumba, 2018) *L. jensenii* (Audirac-Chalifour et al., 2016) *L. gasseri* (Mitra et al., 2016b) Kwasniewski et al. (2018) reported the next list about *Streptococcus* species: *S. agalactiae*, *S. alactolyticus*, *S. anginosus*, *S. australis*, *S. bovis*, *S. cristatus*, *S. fryi*, *S. gallinaceus*, *S. gordonii*, *S. infantis*, *S. intermedius*, *S. macedonicus*, *S. milleri*, *S. mutants*, *S. oligofermentans*, *S. oralis*, *S. oligofermentans*, *S. oralis*, *S. orisratti*, *S. parasanguinis*, *S. pasteuri*, *S. pseudopneumoniae*, *S. sanguinis*, *S. thermophiles*, *S. tigurinus*, *S. vestibularis*	*Fusobacterium* spp. (Audirac-Chalifour et al., 2016; Castanheira et al., 2021) *Sneathia* (Audirac-Chalifour et al., 2016; Castanheira et al., 2021) *G. vaginalis* (Kwasniewski et al., 2018) Kwasniewski et al. (2018), reported the next list about *Streptococcus* species: *S. agalactiae*, *S. alactolyticus*, *S. anginosus*, *S. australis*, *S. bovis*, *S. cristatus*, *S. fryi*, *S. gallinaceus*, *S. gordonii*, *S. infantis*, *S. intermedius*, *S. macedonicus*, *S. milleri*, *S. mutants*, *S. oligofermentans*, *S. oralis*, *S. oligofermentans*, *S. oralis*, *S. orisratti*, *S. parasanguinis*, *S. pasteuri*, *S. pseudopneumoniae*, *S. sanguinis*, *S. thermophiles*, *S. tigurinus*, *S. vestibularis*	*Fusobacterium* spp. (Audirac-Chalifour et al., 2016; Castanheira et al., 2021; Wu et al., 2021) *Sneathia* (Audirac-Chalifour et al., 2016; Castanheira et al., 2021; Wu et al., 2021) *Gardnerella* (Kwasniewski et al., 2018) *G. vaginalis* (Kwasniewski et al., 2018; So et al., 2020; Castanheira et al., 2021) *Prevotella* (Wu et al., 2021) *P. buccalis*, *P. timonensis* (So et al., 2020) *F. vaginae* (So et al., 2020) *Dialister* (So et al., 2020) *Megasphaera* (Audirac-Chalifour et al., 2016; Wu et al., 2021) Kwasniewski et al. (2018), reported the next list about *Streptococcus* species: *S. agalactiae*, *S. alactolyticus*, *S. anginosus*, *S. australis*, *S. bovis*, *S. cristatus*, *S. fryi*, *S. gallinaceus*, *S. gordonii*, *S. infantis*, *S. intermedius*, *S. macedonicus*, *S. milleri*, *S. mutants*, *S. oligofermentans*, *S. oralis*, *S. oligofermentans*, *S. oralis*, *S. orisratti*, *S. parasanguinis*, *S. pasteuri*, *S. pseudopneumoniae*, *S. sanguinis*, *S. thermophiles*, *S. tigurinus*, *S. vestibularis*	*Fusobacterium* (Audirac-Chalifour et al., 2016) *Sneathia* (Mitra et al., 2016b; Sims et al., 2021) *Sneathia sanguinegens* (Mitra et al., 2016a; So et al., 2020) *Mycoplasma* (Klein et al., 2020a) *Chlamydia trachomatis* (Zhou et al., 2021) *Anaerococcus tetradius* (Mitra et al., 2016b) *Peptostreptococcus anaerobius* (Mitra et al., 2016b; So et al., 2020) *Gardnerella* (Kang et al., 2021) *Gardnerella vaginalis* (So et al., 2020; Zhou et al., 2021) *P. bucalis*, *P. timonensis*, *P. disiens* (So et al., 2020) *Fannyhessea vaginae* (Audirac-Chalifour et al., 2016; So et al., 2020) *Streptococcus* (Kang et al., 2021) *Finegoldia magna* (So et al., 2020)	*Fusobacterium* (Sims et al., 2021; Wu et al., 2021) *Fusobacterium necrophorum* (Audirac-Chalifour et al., 2016; So et al., 2020; Castanheira et al., 2021) *Sneathia* spp. (Mitra et al., 2016b) *Sneathia amnii* (Audirac-Chalifour et al., 2016; Mitra et al., 2016b) *Mycoplasma* (Wu et al., 2021) *Anaerococcus* (Kang et al., 2021; Wu et al., 2021) *Peptostreptococcus* (Wu et al., 2021) *P. anaerobius* (So et al., 2020) *G. vaginalis* (Mitra et al., 2016b; So et al., 2020) *Prevotella* (Wu et al., 2021) *P. bucalis*, *P. timonensis* (So et al., 2020) *Fannyhessea vaginae* (So et al., 2020) *Streptococcus* (Audirac-Chalifour et al., 2016; Kang et al., 2021) *Dialister* (Wu et al., 2021) *D. invisus* (So et al., 2020) *Megasphaera* (Audirac-Chalifour et al., 2016; Wu et al., 2021) *Peptoniphilus* (So et al., 2020; Wu et al., 2021) *Finegoldia magna* (So et al., 2020)	**PID:** *P. anaerobius* (So et al., 2020) **HPV:** *Sneathia* spp. (Mitra et al., 2016a,b; Zhou et al., 2021), *Fusobacterium* (Audirac-Chalifour et al., 2016), *Chlamidia trachomatis* (Klein et al., 2020a; Zhou et al., 2021) *Anaerococcus* (Wu et al., 2021), *Gardnerella vaginalis* (Zhou et al., 2021), *Prevotella* (So et al., 2020; Wu et al., 2021), *Streptococcus* (Kang et al., 2021), *Peptoniphilus* (Kang et al., 2021) **BV:** *Fusobacterium* (Amabebe and Anumba, 2018), *Fannyhessea* (Amabebe and Anumba, 2018), (Kwasniewski et al., 2018; Klein et al., 2020a; Castanheira et al., 2021; Zhou et al., 2021), *Mycoplasma* (Amabebe and Anumba, 2018; Kwasniewski et al., 2018; Klein et al., 2020a), *M. genitalium*, *M. hominis* (Klein et al., 2020a; Zhou et al., 2021), *S. sanguinegens* (Klein et al., 2020a), *S. amnii* (Audirac-Chalifour et al., 2016), *Peptostreptococcus* (Mitra et al., 2016b; So et al., 2020), *Gardnerella* (Mitra et al., 2016b), *G. vaginalis* (Klein et al., 2020a; Castanheira et al., 2021), *Prevotella* (Ravel et al., 2011; Amabebe and Anumba, 2018; Kwasniewski et al., 2018; Castanheira et al., 2021; Wu et al., 2021), *Streptococcus* (Amabebe and Anumba, 2018), *Dialister* (Amabebe and Anumba, 2018), *Megasphaera* (Amabebe and Anumba, 2018; Klein et al., 2020a; Castanheira et al., 2021), *Mobiluncus* (Mitra et al., 2016b; Amabebe and Anumba, 2018; Kwasniewski et al., 2018), *M. mulieris*, *M. curtisii* (Klein et al., 2020a)

HC, healthy controls; SIL, squamous intraepithelial lesions; LSIL, low-grade squamous intraepithelial lesions; HSIL, high-grade squamous intraepithelial lesions; CIN, cervical intraepithelial neoplasia; ICC, invasive cervical cancer; CAN, cervical cancer; HPV, human papillomavirus; BV, bacterial vaginitis; PID, pelvic inflammatory disease.

## 5 Microbial markers and cervical cancer

Advancements in microbiome research have unveiled new avenues for understanding the root causes of various diseases, including cancer. With the advent of high-throughput technologies such as genomics, transcriptomics, metagenomics, and metatranscriptomics, researchers can now generate an enormous amount of data (Wei et al., 2021). When it comes to cervical cancer, this vast repository of data is meticulously scrutinized to identify potential biomarkers that could transform its diagnosis and prognosis (Norenhag et al., 2020; Han et al., 2021; Kang et al., 2021; Wei et al., 2021; Zhou et al., 2021).

Researchers propose that certain microorganisms serve as beneficial diagnostic markers for cervical cancer or as indicators of infection severity. From the host’s perspective, diverse types of biomarkers (prognostic, predictive, and diagnostic) are being explored to enhance the management of cervical cancer.

High-throughput technologies have paved the way for a deeper exploration of the complex relationship between microbiota and cancer. The capacity to examine the entire microbiome and its intricate micro-ecosystems has led to the identification of specific microbial entities as predictive markers of cancer (Wei et al., 2021). At present, research is primarily centered around four main areas: characterizing microbial diversity and composition, conducting microbial functional analyses, predicting biomarkers, and investigating potential therapeutic applications. However, these areas are still nascent and need to be solidified in clinical practice (Wei et al., 2021).

To fully unravel the correlation between the microbiome and cancer, the consistent use of high-throughput methodologies is deemed necessary. Various studies conducted on the microbiota associated with cervical cancer or cervical disease have reiterated the urgent need for reliable biomarkers to improve the diagnosis of cervical cancer or prevent it. There is a pressing need to devise novel diagnostic strategies incorporating microbiological markers for early detection of cervical cancer in patients (Kang et al., 2021; Sims et al., 2021; Wei et al., 2021; Wu et al., 2021; Zhou et al., 2021).

Several microorganisms, including *Fusobacterium* spp., *Sneathia* spp. (*S. amnii*), *Anaerococcus* spp., *Peptostreptococcus* spp., *Gardnerella* spp. (*G. vaginalis*), *Prevotella* spp., *Dialister* spp., *Fannyhessea* spp. (*F. vaginae*), *Streptococcus* spp., *Megasphaera* spp., *L. crispatus*, and *L. gasseri* (Kori and Arga, 2018; Klein et al., 2020b; Tango et al., 2020), have been suggested as microbiological markers for cervical cancer. The profound implications of these findings for the future of cancer diagnostics underscore the importance of continued research in this promising field.

## 6 Data exploration

In an effort to gain deeper insights from the literature, a rigorous process of search was carried out. This process targeted papers that provided accessible information in the NCBI databases. Initially, six potential articles were identified, each providing specific details on vaginal microbiota and cervical health conditions, as illustrated in Table 3. However, upon further exploration for raw data within the NCBI, only four of these articles—marked with asterisk—offered the required information.

**Table 3:** Potential articles containing information on vaginal microbiota and cervical health conditions.

Objective	Type of analysis	Technique	Reference
To assess the alteration in vaginal microbiota during the progression of cervical cancer in women infected with high-risk HPV	Metagenomics: 16S rRNA genes	Next-generation sequencing	So et al. (2020)^*^
To define the changes in the cervical microbiome in women of reproductive age during the transition from squamous intraepithelial lesions (SIL) to cervical cancer (CAN)	Metagenomics: 16S rRNA genes	Whole Genome Sequencing	Wu et al. (2021)
To examine the correlation between infections in the cervix and vagina and the development of pre-cancerous cervical lesions	Metagenomics: 16S rRNA genes	Next-generation sequencing	Kwasniewski et al. (2018)
To delve into the possible connection between the composition of vaginal microbes and CAN, presenting its diagnostic value in predicting, classifying, and tracking CAN progression. This also includes differentiating samples from diseased individuals from those of healthy controls	Metagenomics: 16S rRNA genes	High-throughput sequencing	Kang et al. (2021)^*^
To foster a comprehensive and precise comprehension of the structure and ecology of the vaginal microbial ecosystem in women without symptoms, with a focus on understanding the purpose and fundamental operation of the vaginal microbiota	Metagenomics: 16S rRNA genes	Pyrosequencing	Ravel et al. (2011)^*^
To investigate the relationship between the diversity and composition of cervical microbiota, as per a histopathological diagnosis at each stage of CAN’s natural history, and the expression levels of cytokines in the cervix	Metagenomics: 16S rRNA genes	Sanger sequencing	Audirac-Chalifour et al. (2016)^*^

^*^Articles with raw data freely available for analysis.

Despite the heterogeneity inherent in each database, we undertook a data exploration process to confirm if earlier published data (Ravel et al., 2011; Audirac-Chalifour et al., 2016) aligns with recent publications (So et al., 2020; Kang et al., 2021). It is worth noting that such analyses invariably encounter limitations rooted in the data source, standardization of metadata information, and the procedures employed for sequencing results, among other factors. Nevertheless, our investigation focused on deciphering microbiota patterns across various cervical health conditions.

Regarding the fourth study under scrutiny (Kang et al., 2021), the absence of raw data within the manuscript necessitated a comprehensive reanalysis of the samples provided, guided solely by accession numbers for sequences in the NCBI database. This re-evaluation was executed employing the QIIME-2022.8 pipeline, strictly adhering to the author’s guidelines delineated within their paper. Denoising was performed utilizing DADA2 (Divisive Amplicon Denoising Algorithm 2) (Callahan et al., 2016), and despite Kang et al. (2021) usage of the Silva v138 database, we elected to use the Silva (16S/18S rRNA) (Quast et al., 2013; Yilmaz et al., 2014) database v132. Similar to the authors, the sequences among the reanalyzed samples were rarefied to a sequencing depth of 6,919 reads.

Subsequent to the successful acquisition of data from each of the four papers, the next step was to distill this data, as showed in Table 4. All data abundances were normalized to values ranging from 0 to 1. We successfully compiled a total of 496 samples, encapsulating information pertaining to the type of cervical health condition (CAN, CIN, SIL, and control), the respective study reported, and HPV presence (restricted to CAN and Control samples). The statistical analysis and graphic representation were executed using the R 4.2.1 version.

**Table 4:** Data derived from databases on NCBI with accessible raw information (Ravel et al., 2011; Audirac-Chalifour et al., 2016; So et al., 2020; Kang et al., 2021).

Type	*n*	Study	*n*	HPV	
				Positive	Negative
CAN	26	Audirac-Chalifour et al. (2016)	8	8	N/A
		Kang et al. (2021)	8	8	N/A
		So et al. (2020)	10	10	N/A
CIN	28	Kang et al. (2021)	8	N/A	N/A
		So et al. (2020)	20	N/A	N/A
SIL	4	Audirac-Chalifour et al. (2016)	4	N/A	N/A
Control	438	Audirac-Chalifour et al. (2016)	17	10	7
		Kang et al. (2021)	7	N/A	7
		So et al. (2020)	20	10	10
		Ravel et al. (2011)	394	N/A	394
Total	496				

*n*, number of samples.

It is important to highlight that our analysis was conducted within certain constraints. The availability of raw data posed a significant limitation, necessitating the reanalysis of samples in specific instances. Additionally, the low number of articles utilized for our analysis, owing to our commitment to use only freely accessible information, may have affected the comprehensiveness of our study. Despite these challenges, we remained committed to conducting a meticulous and robust exploration of the available data.

According to the previous Table 1, where is presented the microorganisms proposed as microbiological markers in cervical cancer or cervix inflammation, the common bacteria genera found in each one of the four articles that were also mentioned in this table (as potential biomarkers) were: *Fusobaterium* spp., *Sneathia* spp., *Streptococcus* spp., *Gardnerella* spp., *Dialister* spp., *Megasphaera* spp., *Peptostreptococcus* spp., *Peptoniphilus* spp., *Prevotella* spp., *Anaerococcus* spp., and *Lactobacillus* spp.

**Figure 2:**
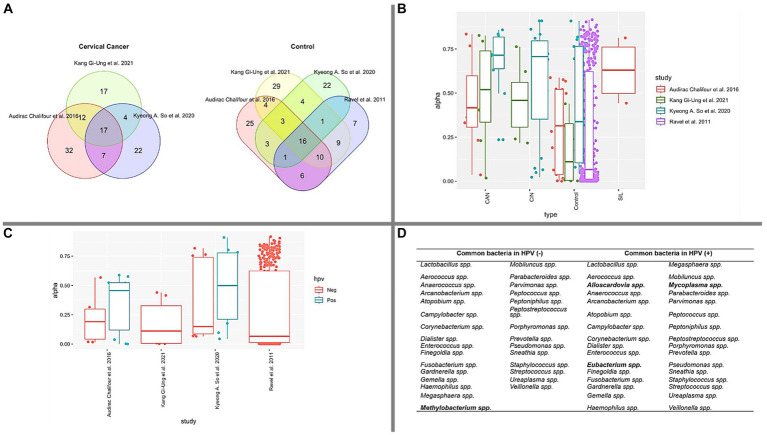
Bacterial composition and diversity. **(A)** The Venn diagram depicts the overlap of common bacterial types identified in CAN and CONTROL groups, based on the 50 most abundant bacteria in each study. **(B)** Simpson’s index measures genera diversity across various cervical health conditions. Notably, CONTROL samples exhibit lower diversity compared to CAN, CIN, and SIL conditions. The Kruskal-Wallis test yielded a *p*-value <0.05, signifying significant differences in diversity between CONTROL vs. CAN and CONTROL vs. CIN. **(C)** A box plot illustrating the prevalence of HPV infection in control samples across the studied papers. **(D)** Identification of common bacterial types in HPV (−) and HPV (+) groups in each paper, with unique bacteria within each group highlighted in bold.

Our findings, as outlined in Table 4, consistently demonstrate the presence of HPV in all documented cases of cervical abnormalities (CAN). To gain a deeper understanding of the bacterial profile associated with CAN, we employed a Venn diagram, as illustrated in Figure 2A. This visualization not only highlights the commonly identified bacteria in CAN cases, but also those observed in control samples. In the context of CAN, our analysis revealed 17 frequently reported bacterial genera: *Fusobacterium* spp., *Sneathia* spp., *Streptococcus* spp., *Gardnerella* spp., *Dialister* spp., M*egasphaera* spp., *Peptostreptococcus* spp., *Peptoniphilus* spp., *Staphylococcus* spp., *Campylobacter* spp., *Parvimonas* spp., *Prevotella* spp., *Haemophilus* spp., *Porphyromonas* spp., *Anaerococcus* spp., *Lactobacillus* spp., *Ureaplasma* spp.

To further elucidate the bacterial landscape, an additional Venn diagram was constructed to identify common bacterial genera in control samples from the studies examined (Figure 2A). We observed that 16 genera were common in these samples: *Sneathia* spp., *Streptococcus* spp., *Gardnerella* spp., *Dialister* spp., *Megasphaera* spp., *Peptoniphilus* spp., *Staphylococcus* spp., *Parvimonas* spp., *Prevotella* spp., *Porphyromonas* spp., *Anaerococcus* spp., *Lactobacillus* spp., *Ureaplasma* spp., *Aerococcus* spp., *Finegoldia* spp., *Enterococcus* spp.

In reference to Table 1, which proposes certain microorganisms as potential microbiological markers for cervical cancer or cervix inflammation, we noted that the following genera were shared between our four selected articles and those suggested as potential biomarkers: *Fusobacterium* spp., *Sneathia* spp., *Streptococcus* spp., *Gardnerella* spp., *Dialister* spp., *Megasphaera* spp., *Peptostreptococcus* spp., *Peptoniphilus* spp., *Prevotella* spp., *Anaerococcus* spp., and *Lactobacillus* spp. This overlap may indicate a significant link between these bacterial genera and cervical health disorders.

Simpson’s diversity index, a standard tool for determining alpha diversity, gauges the prevalence of dominant species and inversely correlates with species diversity (Sagar and Sharma, 2012). As illustrated in Figure 2B, our analyses calculated this alpha diversity. The data suggests that as the cervical health condition transitions from Control to SIL, CIN, and CAN, there is an observable increase in microbiota, corroborating previous literature (Klein et al., 2020a; Norenhag et al., 2020; So et al., 2020; Tango et al., 2020; Kang et al., 2021; Sims et al., 2021; Wu et al., 2021; Zhou et al., 2021). Figure 2C presents a similar pattern for HPV (−) samples, where bacterial diversity is comparatively lower than HPV (+) samples. The median value for boxes representing HPV (−) samples is less than 0.25, contrasting with those representing HPV (+) samples. Furthermore, we compiled a list of the top 50 bacterial genera present in HPV (+) and HPV (−) samples across the four studied papers. As depicted in Figure 2D, these bacteria are displayed accordingly.

It is crucial to emphasize that our exploration of the data did not follow the strict guidelines of a meta-analysis or systematic review. This absence of a structured approach may introduce a potential bias in our findings, as we might have overlooked certain studies or emphasized others disproportionately. Also, because we focused on information that’s freely available, there may be some limits to the scope and depth of our analysis. Despite these limitations, we see our work as an initial step in understanding the overall trends in microbiota composition during cervical cancer progression. Our findings should be interpreted with caution, considering the potential biases and methodological constraints. However, we believe our research provides valuable insights that can pave the way for future, more thorough investigations in this crucial area.

### 6.1 Microbiota and HPV

Based on the distinctive microorganisms identified solely in HPV (+) and HPV (−) samples (Figure 2D), existing literature has associated *Methylobacterium* spp. as a predominant bacteria in the ovary (Amabebe and Anumba, 2020) and ovarian cancer (Peric et al., 2019). Our data, as presented in Figure 2D, identified *Methylobacterium* spp. within the HPV (−) groups. Notably, this microorganism emerged as the sole differential entity when compared to HPV (+) samples. However, given the inherent limitations of a review paper, such as data heterogeneity, further research is required to substantiate these findings.

Contrarily, *Alloscardovia* spp., *Eubacterium* spp., and *Mycoplasma* spp. were exclusively detected in HPV (+) samples. Previous reports have also documented *Alloscardovia* spp. in HPV (+) samples (Gao et al., 2013), and a 2019 case study associated this microorganism with preterm premature rupture of membranes (PPROM) (Cardona-Benavides et al., 2019). *Eubacterium* spp., meanwhile, has been reported more frequently in HPV (+) patients than in HPV (−) patients (Carrillo-Ng et al., 2021) and is also associated with BV cases (Fredricks et al., 2005; Srinivasan and Fredricks, 2008). Moreover, *Mycoplasma* spp. has been found to be prevalent among women with HPV (+) (Klein et al., 2020b), with its abundance noted to increase in women with cervical lesions (Pacha-Herrera et al., 2022). *Mycoplasma* spp. has also been implicated as a potential cause of persistent HPV infection (Zhou et al., 2021) and has been identified in BV infections (Ferris et al., 2004; Verhelst et al., 2004; Kwasniewski et al., 2018).

Our review suggests that *Alloscardovia* spp., *Eubacterium* spp., and *Mycoplasma* spp. could potentially serve as biomarkers for HPV (+), while *Methylobacterium* spp. might be a marker for HPV (−). However, it’s important to note that due to the limitations inherent in this review and the data evaluated, these observations remain tentative. There is a clear need for continued research to further explore the role of the microbiota in the development of HPV, as this could provide valuable insights that may aid in the fight against this condition.

### 6.2 Microbiota and cervical cancer progression

Based on the findings outlined in Section 6, Table 5 encapsulates the shared bacteria identified in the intersection of the Venn diagram depicted in Figure 2A. This table effectively illustrates the genera of bacteria that appear to be prevalent as cervical cancer progresses, as well as in healthy controls.

**Table 5:** Microbiota and its presence according to the health condition: CAN or CONTROL.

Bacteria	CAN^*^	CONTROL^*^
*Fusobacterium* spp.	X	
*Sneathia* spp.	X	X
*Streptococcus* spp.	X	X
*Gardnerella* spp.	X	X
*Dialister* spp.	X	X
*Megasphaera* spp.	X	X
*Peptostreptococcus* spp.	X	
*Peptoniphilus* spp.	X	X
*Staphylococcus* spp.	X	X
*Campylobacter* spp.	X	
*Parvimonas* spp.	X	X
*Prevotella* spp.	X	X
*Haemophilus* spp.	X	
*Porphyromonas* spp.	X	X
*Anaerococcus* spp.	X	X
*Lactobacillus* spp.	X	X
*Ureaplasma* spp.	X	X
*Aerococcus* spp.		X
*Finegoldia* spp.		X
*Enterococcus* spp.		X

^*^Data according to Figure 2.

Focusing first on the CONTROL samples, *Fusobacterium* spp., *Peptostreptococcus* spp., *Campylobacter* spp., and *Haemophilus* spp. were conspicuously absent from the bacterial genera identified. Drawing from existing literature, *Fusobacterium* spp. has been exclusively reported in CAN or CIN samples, which may account for the numerous propositions of *Fusobacterium* spp. as a potential marker of CAN (Audirac-Chalifour et al., 2016; Norenhag et al., 2020; So et al., 2020; Castanheira et al., 2021; Sims et al., 2021; Wu et al., 2021; Zhou et al., 2021). The potential of *Fusobacterium* spp. as an oncogenic entity and a promoter of dysplasia development has also been deliberated (Norenhag et al., 2020). Additional characteristics of this microorganism are detailed in Table 1. Thus, Table 5 in alignment with the literature, indicates that *Fusobacterium* spp. is only present in CIN, SIL, and CAN samples.

*Peptostreptococcus* spp., a bacterial genus not detected in control samples (refer to Table 5), is associated with cervical conditions such as cervical intraepithelial neoplasia (CIN) and cervical cancer (CAN) (Mitra et al., 2016b; So et al., 2020). This bacterium also plays a role in female genital tract infections like bacterial vaginosis and pelvic inflammatory disease (So et al., 2020) and is considered a distinctive marker for the CAN group (Wu et al., 2021).

*Campylobacter* spp., identified as a CAN marker (Wu et al., 2021), was first reported in CIN cases in 2018 (Zhang et al., 2018). This review’s analysis revealed the presence of this bacterium exclusively in CAN samples (Table 5).

*Haemophilus* spp. is another bacterial genus absent in control samples. It is believed to contribute to trichomoniasis as a colonizing microorganism (Kwasniewski et al., 2018) and has been reported only in CAN samples (So et al., 2020), explaining its absence in the control group (Table 5).

Three bacterial genera—*Finegoldia* spp., *Enterococcus* spp., and *Aerococcus* spp.—were found solely in control samples, contrasting with CAN samples (Table 5). *Finegoldia*’s spp. exclusive presence in control samples might be due to its higher abundance compared to other conditions, as analyses focused on the top 50 most abundant bacteria from each studied paper. More information about *Finegoldia* spp. can be found in Table 1.

*Enterococcus* spp., commonly found in healthy samples and associated with HPV clearance (Verhelst et al., 2004; Zhou et al., 2007; Ravel et al., 2011; Borgdorff et al., 2014; di Paola et al., 2017), aligns with the results observed in Table 5.

*Aerococcus* spp., another genus found exclusively in control samples, corroborates previous literature (Verhelst et al., 2004; Zhou et al., 2007; Srinivasan and Fredricks, 2008; Ravel et al., 2011). Additional information about *Aerococcus* spp. is available in Table 1.

*Sneathia* spp., *Streptococcus* spp., *Gardnerella* spp., *Dialister* spp., *Megasphaera* spp., *Peptoniphilus* spp., *Staphylococcus* spp., *Parvimonas* spp., *Prevotella* spp., *Porphyromonas* spp., *Anaerococcus* spp., *Lactobacillus* spp., and *Ureaplasma* spp. were found in both CAN and control samples.

*Sneathia* spp. has been reported in CIN samples (So et al., 2020), bacterial vaginosis (BV) cases (Srinivasan and Fredricks, 2008; Liu et al., 2013; Borgdorff et al., 2014; Klein et al., 2020a; Zhou et al., 2021), squamous intraepithelial lesion (SIL) samples (Audirac-Chalifour et al., 2016; Wu et al., 2021), HPV infections (Audirac-Chalifour et al., 2016; Dareng et al., 2016; di Paola et al., 2017; Castanheira et al., 2021; Zhou et al., 2021) and as a biomarker of cervical neoplasia (Godoy-Vitorino et al., 2018). However, it is also a common member of the vaginal community (Verhelst et al., 2004; Verstraelen et al., 2004; Kang et al., 2021), suggesting it should not be considered a biomarker. More information about *Sneathia* spp. is provided in Table 1.

*Streptococcus* spp. has been observed in CAN samples (So et al., 2020; Tango et al., 2020; Wu et al., 2021), cervical disease cases (So et al., 2020), and CIN patients (Tango et al., 2020; Arroyo Mühr et al., 2021). Still, it was also identified as part of the vaginal composition (Verhelst et al., 2004; Zhou et al., 2007; Srinivasan and Fredricks, 2008; Gao et al., 2013; Audirac-Chalifour et al., 2016; Arroyo Mühr et al., 2021; Kang et al., 2021; Wu et al., 2021; Zhou et al., 2021) and associated with HPV clearance (di Paola et al., 2017), which aligns with our findings that this microorganism appears in both groups. Further information about Streptococcus can be found in Table 1.

*Gardnerella* spp., a bacterium identified in both disease and control samples, is classified as an anaerobic carcinogen (Zhou et al., 2021). It has been found in cervical intraepithelial neoplasia (CIN) samples (Mitra et al., 2016b; So et al., 2020; Sims et al., 2021), bacterial vaginosis (BV) cases (Borgdorff et al., 2016; Mitra et al., 2016b; Amabebe and Anumba, 2018; Klein et al., 2020a; Castanheira et al., 2021), HPV persistent infections (Norenhag et al., 2020), and control samples (Audirac-Chalifour et al., 2016; Tango et al., 2020). This aligns with our paper analysis results. Additional information about Gardnerella can be found in Table 1.

*Dialister* spp., another genus present in both groups (cervical cancer and control), is an opportunistic pathogen influenced by *Lactobacillus* spp. (Wu et al., 2021). It has been reported as a marker genus in cervical cancer (So et al., 2020; Sims et al., 2021; Wu et al., 2021), and found in CIN samples (So et al., 2020), BV (van de Wijgert et al., 2014; di Paola et al., 2017; Amabebe and Anumba, 2020), and HPV cases (Gao et al., 2013; Audirac-Chalifour et al., 2016; Dareng et al., 2016). However, it’s also been observed in control samples (Ravel et al., 2011; So et al., 2020), which concurs with our findings. More details about *Dialister* spp. are provided in Table 1.

*Megasphaera* spp., found in both disease and normal samples, has been reported in squamous intraepithelial lesion (SIL) and cervical cancer samples (Wu et al., 2021), CIN cases (Mitra et al., 2016b; Sims et al., 2021), BV patients (Amabebe and Anumba, 2018; Klein et al., 2020a; Castanheira et al., 2021) and normal samples (Ravel et al., 2011; Arroyo Mühr et al., 2021). Table 1 provides more details on this bacterium.

*Peptoniphilus* spp., another common bacterium, has been reported as a cervical cancer marker (So et al., 2020; Wu et al., 2021), found in BV samples (Fredricks et al., 2005; Srinivasan and Fredricks, 2008; van de Wijgert et al., 2014), HPV infections (Shannon et al., 2017; Kang et al., 2021), and control samples (Srinivasan and Fredricks, 2008; Ravel et al., 2011). Further details about *Peptoniphilus* spp. can be found in Table 1.

*Staphylococcus* spp., although common in control samples (Tango et al., 2020), has also been observed in conditions such as cervical cancer (Tango et al., 2020; Arroyo Mühr et al., 2021), aerobic vaginitis (di Paola et al., 2017), and SIL (Klein et al., 2020a; Arroyo Mühr et al., 2021; Sims et al., 2021; Wu et al., 2021).

*Parvimonas* spp. is another bacterium observed in both cervical cancer and control samples. It has been reported in control samples (Shannon et al., 2017), cervical diseases (Godoy-Vitorino et al., 2018; So et al., 2020), and BV patients (van de Wijgert et al., 2014).

*Prevotella* spp. has been found in control samples (Lewis et al., 2017; Zhou et al., 2021) and other conditions like HPV infections (Norenhag et al., 2020), CIN (Mitra et al., 2016b; Godoy-Vitorino et al., 2018; Tango et al., 2020; Sims et al., 2021), CAN (Wu et al., 2021), and BV (di Paola et al., 2017; Amabebe and Anumba, 2018; Kwasniewski et al., 2018; Castanheira et al., 2021). This aligns with the results summarized in Table 5 and detailed in Table 1.

*Porphyromonas* spp., also present in both disease and control cases (Table 5), has been observed in CAN (Sims et al., 2019, 2021; Wu et al., 2021), BV samples (Fredricks et al., 2005; Srinivasan and Fredricks, 2008; van de Wijgert et al., 2014), and control cases (Verhelst et al., 2004; Srinivasan and Fredricks, 2008).

*Anaerococcus* spp. has been reported in control samples (Verhelst et al., 2004; Zhou et al., 2007; Srinivasan and Fredricks, 2008; Arroyo Mühr et al., 2021), and diseases such as CAN (Wu et al., 2021), SIL (Mitra et al., 2015, 2016b), and CIN cases (Mitra et al., 2016b; Godoy-Vitorino et al., 2018).

*Lactobacillus* spp., another common bacterium (Table 5), is seen in various conditions depending on the species—either as a disease marker or a health biomarker in the vaginal composition. For instance, it has been reported in conditions like CAN (Castanheira et al., 2021), CIN (Mitra et al., 2016b; Norenhag et al., 2020; Sims et al., 2021), SIL (Norenhag et al., 2020), HPV clearance (Mitra et al., 2016b; Norenhag et al., 2020), cervical dysplasia (Norenhag et al., 2020), and control samples (Mitra et al., 2016b; Amabebe and Anumba, 2018; Klein et al., 2020a; Norenhag et al., 2020; Castanheira et al., 2021; Sims et al., 2021; Zhou et al., 2021). More information about different *Lactobacillus* species (*L. jensenii*, *L. gasseri*, *L. crispatus*, *L. inners*) can be found in Table 1.

Lastly, *Ureaplasma* spp. has been reported in both CAN (Tango et al., 2020) and control samples (Verhelst et al., 2004; Srinivasan and Fredricks, 2008; Wu et al., 2021), as well as in CIN (Tango et al., 2020), and BV patients (Fredricks et al., 2005; van de Wijgert et al., 2014; Amabebe and Anumba, 2018; Klein et al., 2020a).

The exclusive presence of specific microorganisms in cervical adenocarcinoma cases such as *Fusobacterium* spp., *Peptostreptococcus* spp., *Campylobacter* spp., and *Haemophilus* spp., underscores their potential significance in the pathology of this disease. These unique bacteria could play important roles in the onset and progression of CAN, and their further investigation may provide valuable insights for the development of new diagnostic markers or therapeutic strategies. Understanding the specific functions and influences of these bacteria in CAN is a crucial step toward improving our ability to prevent and treat this form of cervical cancer.

## 7 Discussion

The evolution of sequencing methodologies has paved the way for revolutionary advancements in our understanding of microbiomes and associated diseases, including cervical cancer. This progression can be traced through the various technological tools utilized in the quest for comprehension.

In this context, innovative technologies like NextSeq500 (by Illumina) have been employed for parallel DNA and RNA sequencing to comprehensively detect detectable and actively transcribed DNA and RNA microbes in cervical specimens. The results from such studies suggest that the choice of approach (RNA-Seq, DNA-Seq) can influence the number of transcripts obtained. The focus of current research endeavors is to maximize sequence retention in order to amass a wealth of data that could prove beneficial for multiple investigations reliant on database information.

These technological advancements present an exciting opportunity to delve deeper into the intricacies of cervical cancer and its relationship with the microbiota. The wealth of information that these technologies can provide will undoubtedly fuel further research, and potentially lead to breakthroughs in diagnostic and therapeutic strategies.

The analyses conducted herein strongly advocate for continued biomarker exploration and the need for up-to-date data to inform the development of new strategies to combat cervical cancer.

Our review provides a comprehensive overview of the microbiota associated with the progression of cervical cancer and enumerates several microorganisms implicated in cervicovaginal dysbiosis.

Furthermore, we outline the principal discoveries of past research related to the microbiota present during the CONTROL (healthy) and CAN stages, as well as other conditions such as CIN, SIL, HPV (+), and HPV (−). This analysis allowed us to identify promising bacteria frequently reported as biomarkers, suggesting that biomarker identification is a compelling field with potential for numerous research projects.

A consistent presence of HPV was demonstrated in all reported cases of cervical abnormalities. We have identified noteworthy bacterial genera that differ between both CAN and control samples through our investigation. The intersection of these identified bacteria with those suggested as microbiological indicators for cervical health issues in existing research implies a potential connection that needs additional exploration. This understanding of the microbial landscape may provide valuable insights into the pathogenesis of cervical diseases and potentially guide future diagnostic and prevention strategies and treatment plans.

The advent of sequencing techniques has shed new light on our understanding of microbial biomarkers. The rise of this technology holds the promise of facilitating more in-depth studies examining the relationship between cancer and the microbiome. Nevertheless, there is a pressing need for additional research and the standardization of methods for metadata acquisition. This will enhance the scalability of results, ultimately aiming to positively impact the health and wellness of women worldwide.

## Author contributions

WF: Conceptualization, Investigation, Methodology, Visualization, Writing – original draft, Writing – review & editing. PA: Conceptualization, Project administration, Supervision, Visualization, Writing – review & editing. LV: Visualization, Writing – review & editing. FM: Investigation, Methodology, Project administration, Supervision, Visualization, Writing – review & editing.
